# The Role of Chinese Language Learners' Academic Resilience and Mindfulness in Their Engagement

**DOI:** 10.3389/fpsyg.2022.916306

**Published:** 2022-06-02

**Authors:** Wei Liu, Yonggang Gao, Lu Gan, Jinwen Wu

**Affiliations:** ^1^School of Foreign Languages, Hubei University of Education, Wuhan, China; ^2^School of Foreign Languages, China University of Geosciences Wuhan, Wuhan, China

**Keywords:** academic resilience, mindfulness, academic engagement, Chinese language learners, positive psychology

## Abstract

One of the growing area of interest in the educational area is student engagement which is the major construct of positive psychology (PP) vital in growing energetic, innovative, and pleasurable learning, but unluckily, all students are not engaged in terms of cognition, emotion, and behavior in learning. Another concept in the PP literature is resilience which emphasizes institutes' and people's powers and self-constraint to conform to accidental conditions. Furthermore, mindfulness as a significant term in PP has critical benefits such as improving working memory, improving wellbeing, and lowering tension. Considering the importance of mindfulness and engagement in academic environments and that such a notion in foreign language learning is neglected, the current study attempts to inspect the effect of mindfulness and resilience on the engagement of Chinese foreign language students. To meet this objective, 1,693 EFL learners participated in this study. They responded to the mindfulness scale, resilience scale, and engagement questionnaire. Subsequently, the Spearman Rho test was exploited to shed light on probable relationships. The findings indicated that there was a significant correlation among the variable of the study. Moreover, a linear multiple regression analysis was run to examine the predictor roles of mindfulness and resilience in learners' engagement. The findings revealed that both mindfulness and resilience are positive and reliable predictors of engagement. In a nutshell, the central position of resilience and mindfulness in language learning was verified, and based on the findings; a few suggestions are made considering the results of the research.

## Introduction

Students usually have tension and apprehension about the school, social environment, future ambiguity, and different variables like future jobs (Rew et al., 2014). The tension of learners is associated with a myriad of unpleasant results that encompass poor educational performance, executive performance shortages, the enhanced danger of maladaptive manners, and low satisfaction with life (Agoston and Rudolph, 2016). In addition, people who fail to deal with academic challenges are exposed to different unfavorable affections, namely, tension, apprehension, anxiety, and fatigue, which all can lead to their disengagement (Xie, 2021). As the symptoms of disengagement start in learners' lives, either in or out of school, adults can interfere to aid the learner to cope with the affective or development difficulties in a way that causes the development of engagement (Sulkowski et al., 2012). The degree of interest, concentration, optimism, passion, inquisitiveness, or incentive learners experience while learning or teaching is called engagement (Derakhshan et al., 2022); and it is characterized as multidimensional, regularly evaluated based on what learners do, think, and feel that have a straightforward relation with education (Fredricks et al., 2019). Learners' engagement is significant in educational activities because learners should dynamically take part in the educational cycle (Artika et al., 2021) and it can be witnessed through learners' focus, attractiveness, and ease in attaining education (Widyaswara et al., 2019), and it is taken into consideration as a pretty suitable and crucial precondition of learning in EFL classrooms (Dörnyei, 2019). Engaging in education is lively and self-motivated and is likely to have favorable outcomes in the educational presentation (Reschly and Christenson, 2012). It is crucial to comprehend the intellectual, affective, and behavioral elements when deciding the level of English educational engagement to advance the effective achievement of English in an EFL setting (Dincer et al., 2019). As intellectual elements related to engagement, mindful training is being debated has been at the center of attention as an element of the conceptual framework (Schreiner and Louis, 2006). The engagement of students is a vital issue in academic practices which implies students' incentive and energetic engagement in centric programs of academic organization (Mercer, 2019; Wang et al., 2021).

Moreover, even though learners encounter stressing factors in life in a large number of spheres that damages wellbeing improvement, resilience is an essential set of personal tendencies that may function as a protecting mechanism in helping people to triumph and succeed in difficult situations (Ayyash-Abdo et al., 2016). Furthermore, resilience is a significant element in stopping the growth of psychopathology and retaining maximum functioning and physical and mental health regardless of anxiety-provoking life conditions (Ryff and Singer, 2003). As stated by Fletcher and Sarkar (2013), over the last 20 years, different meanings for resilience have been suggested with great inconsistencies throughout the literature. In spite of the definitional and conceptual inconsistencies, most meanings are founded revolving around the two main notions of difficulty and constructive adjustment (Windle et al., 2011). The notion of resilience has newly been characterized as the capability of adjusting and not breaking, getting back up, and even growing when encountering demanding life practices (Southwick et al., 2014). Therefore, required states for resilience to take place include difficulties, disasters, jeopardies, great amounts of anxiety, and other harmful risk in the school setting that each of them impacts educational advancement (Agasisti et al., 2018). Positive consequences pertaining to resilience are the mitigation of the terrible tension effects, the improvement of adaptation, and the improvement of efficient managing competencies to address alterations and difficulties (Ahern et al., 2006). Numerous scholars maintain that resilience may be reinforced as it is not always a “hard-wired” character feature that only a few have and is the outcome of the growth of shielding elements (Reivich and Shatte, 2002). Consequently, reinforcing the protective issues related to resilience will reinforce it and alternatively, learners need to grow mental features to facilitate coping with tension that also improves their engagement. Mindfulness is a mental feature allowing people to conquer experiences of tension and enhance health (Chen and Murphy, 2019). Moreover, a literature review showed a mindfulness-resilience relationship as resilience can be learned through mental interventions and it is also stated that teaching based on mindfulness can be an effective interference for growing resilience (Lightsey, 2006).

Mindfulness can be characterized as the cycle of focusing in particular ways like recognizing, concentrating on the current state, and being unbiased (Kabat-Zinn, 2013) and it has five dimensions of monitoring, explaining, performing with recognition, accepting without forming an opinion, and not being responsive (Baer et al., 2004). Thus, it has the role of personal recognition that assists people with eliminating automatic beliefs and unhealthy conducts and the skill of controlling their conduct (Bajaj et al., 2016). Indeed, mindfulness represents a competency that improves adaptive managing the stressing conditions through self-regulating concentration toward the instant experience, and free and admitting inclination to individuals' experience of the current time (Bishop et al., 2004). Thinking and feelings that get into a person's attention are observed and are not extended on or assessed, with concentration reoriented to breathing in the current time (Chiesa and Malinowski, 2011). Mindfulness promotes focus on and recognition of experiences that occur in the current time (Kabat-Zinn, 2013). In fact, it means the ability to pay attention and getting aware of what is currently taking place (Brown and Ryan, 2003). Awareness is the capability of overseeing the internal and external setting and surrounding continuously; that is, it is the background radar of awareness. Focus alludes to the capability of concentrating on the awareness of one's restricted experience (Brown and Ryan, 2003). Focus and recognition underpin engagement, which is one of the fundamental sub-elements of mindfulness (Kabat-Zinn, 2013). The greater the degree of mindfulness, the more the possibility of flow experiences, and the more significant the individual engagement.

As stated by Minkos et al. (2018), mindfulness can be successful in elevating learners' educational engagement and it plays the role of an approach that assists learners with better recognize educational circumstances, thereby enabling them to be more engaged in education (Lin, 2020). Experiential proof of studies on mindfulness and learner engagement is lacking. Thompson et al. (2011) have declared that mindfulness is related to resilience, and these outcomes propose that mindfulness demonstrates the possibility of being a protective element that increases resilience. For instance, a study by Jha et al. (2010) supported the idea of a relationship between mindfulness and resilience. However, so far there are not enough studies supporting the predictive capabilities of resilience and mindfulness on engagement. Consequently, the goal of the present paper is to scrutinize the relationship between mindfulness, resilience, and engagement and their probable influence on engagement.

## Literature Review

### Student Engagement

Within PP, engagement refers to high interest, immersion, or attention in daily activities (Wang et al., 2022). In such conditions, people are completely involved and engaged in tasks and use their interests and capabilities to analyze. High levels of engagement in tasks refer to a process or the overall experience of struggling (Seligman, 2018). This takes place within the classroom or whenever students engage in their educational tasks such as studying. Educational engagement is a constructive and emotional-cognitive status of mental health featuring power, commitment, and attraction (Schaufeli and Bakker, 2004). Students' engagement is significantly effective in academic psychology regarding educational fulfillment and intrinsic motivation (Salanova et al., 2010). It is also flexible regarding inspiration and constructive educational results and can be elevated as a reaction to intervention (Furlong and Christenson, 2008). Learner engagement can come before educational success or be a result of educational success, which can make it particularly significant for weak or endeavoring learners (Reschly and Christenson, 2012). Advancing learners' engagement ends in better results like educational achievement, constructive self-concept, better career possibilities, greater degrees of life fulfillment, and overall wellbeing for learners specifically among racial and cultural minorities, learners with immigrant guardians, learners living in low SES families, and in poor societies (Wang et al., 2021).

Learners' engagement is a significant concept; however, it is unclear how one can characterize, operationalize, and estimate it. It is multifaceted and intricate, combining learners' thoughts, emotions, and conducts (Furlong and Christenson, 2008). Most models of learners' engagement incorporate behavioral, emotive/affective, and intellectual engagement subcategories. Some include elements like intellectual engagement, which overlaps with behavioral engagement; inspirational engagement, which attends to elements of emotive engagement; and/or mental engagement, which substitutes emotive or intellectual engagement, based on the model (Upadyaya and Salmela-Aro, 2013). Due to the absence of clearness and the important overlap of concepts between the models utilized in the study, the scholar utilizes school engagement, learner engagement in or with school, and learner school engagement alternately based on the best contextual fit.

Overall, behavioral engagement is measurable by evaluating learners' frequency of endeavor, attendance, focus, perseverance, and asking for assistance (Linnenbrink and Pintrich, 2003). Intellectual engagement alludes to the thought cycle, the standard of endeavor, and the skill of self-observing and assessment techniques (Lei et al., 2018). Emotive engagement alludes to being attached to educators and colleagues and having emotions toward the school and education (Hazel et al., 2013). Affective engagement alludes to a sense of belonging and having a rapport with the school. Inspirational engagement includes interest, worth, and affect, which bring about investment in education (Lei et al., 2018). Even though behavioral engagement is the most externally observed and where studies have emphasized, it is not a dependable predictor of learners' engagement on its own (Linnenbrink and Pintrich, 2003). Attributes of engaged learners are as follows: possessing a constructive emotive mood, going to class, wanting to take part in education and maintaining participation in the educational activity, taking action when given the chance, listening to educators' orders, possessing a liking for carrying out tasks, handing in assignments, having extreme endeavor and focus, persevering when the task becomes difficult, choosing assignments at the border of capacity, and enjoying successes (Fletcher, 2013).

### Resilience

The construct “resilience” alludes to inferences that some experience rather good mental results despite being exposed to severe or long-term inconveniences related to deconstructive results (Rutter, 2006). Moreover, it is regularly characterized in dictionaries as the skill of bouncing back swiftly from difficulties or an item or material's capability of going back to its initial shape after being bent or stretched (Lin et al., 2013). Besides these meanings, the word resilience has nine other complex undertones in psychology than what is seen in dictionaries. Studies on resilience began at the beginning of the 1970s when the notion was initially presented in psychology, employing unintentional outcomes from studies in psychiatry and progressive psychology (Luthar, 2006). From its beginning in psychology, resilience has been characterized in different ways because of the absence of an agreement among researchers with various study emphases and methods (Fletcher and Sarkar, 2013). It is the procedure, capability, and the result of an effective adjustment in spite of difficult or jeopardizing situations, and the skill of resisting, adjusting to, and bouncing back from difficulty and anxiety (Borman and Overman, 2004). In an educational setting, resilience may feature learners who can cope with educational difficulties and failure when other learners have a poor function or even experience failure (Martin and Marsh, 2006).

Based on Cassidy (2015), educational resilience features three elements, namely, persistence, thinking and compatible search for help, and negative effect and affective reaction (Cassidy, 2015). Persistence means perseverance that is, while learners enjoy educational resilience, they should be diligent when encountering their educational problems. Thinking and compatible search point to learners' capability to think about their capabilities so that they search for help adaptively based on their capabilities. Such factors include the capability to think about the learners' powers (Cassidy, 2015). The third factor is the negative effect on an affective reaction. This factor describes how the impact of unfavorable events can then bring about an affective reaction in learners. Moreover, it involves descriptions pertaining to the control of apprehension and unfavorable conditions. For an instance, whilst learners enjoy high educational resilience capabilities, they could prevent prolonged affective reactions and stay positive about the problems they encounter (Cassidy, 2015). Learners with educational resilience have been demonstrated to have good relational abilities, self-esteem regarding their capability of studying, constructive demeanor toward the school, honor of culture, and great expectations (Borman and Overman, 2004).

### Mindfulness

Mindfulness is characterized as a condition of focusing on and recognizing what is taking place in the current moment (Brown and Ryan, 2003). To exercise mindfulness, a person should utilize a group of multifaceted abilities like being focused on the current moment and possessing unbiased recognition (Barnes and Lynn, 2010). These conducts give perception, assist with focusing, and offer advantages that come along with mental relaxation (Rosenzweig et al., 2003). Mindfulness is related to feelings of power and self-respect people with high levels of mindfulness are commonly expected to have a higher thinking-feeling adaptation and increase their self-efficiency (Hosseinzadeh et al., 2019). The concept of mindfulness implies a trait and a situation that may be constructed thru practicing (Brown et al., 2007) and it consists of two essential systems: self-regulation of concentration and impartial awareness of experience (Bishop et al., 2004). The first motivates awareness of the emotional, mental, and bodily encountering that usually happens, and the second, which is explained by open-mindedness, interest, and acceptance of that encounter can empower management through decreasing reactiveness. Mindfulness interferences have the potential to elevate constructive affection, positivity, societal emotive capability, and a developing way of thinking, in addition to transitioning intellectual processing toward a greater skill of handling deconstructive feelings and inconvenience that could emerge when addressing novel educational content (Sanger and Dorjee, 2015). At the University of California, a study was led in which learners had a greater result when they had engaged in mindfulness as opposed to the past result before the mindfulness exercise (Docksai, 2013). This happened since mindfulness helps learners enhance their self-control and their awareness of self-effectiveness, which decides learners' educational achievement (McCloskey, 2015). Based on previous studies, mindfulness exercises can reduce anxiety, enhance constructive mood, lessen deconstructive mood, and improve societal, emotive, behavioral, and physical health results (Black, 2015). Furthermore, mindfulness can enhance the recognition of one's thoughts, emotions, and actions, and enhance focus, intellectual regulation, and emotive control (Rempel, 2012; Shonin et al., 2013).

Based on the above-mentioned review of the literature and the research gaps in the literature, the following research questions are proposed:

Q1: Is there any relationship between Chinese EFL students' mindfulness, resilience, and engagement.

Q2: Can Chinese EFL students' mindfulness and resilience predict their engagement?

## Methods

### Participants

The target participants were 1,693 language learners (1,669 valid cases) with different academic qualifications including both genders (male = 1,092, female = 577) whose ages ranged from 13 to 48. They were currently studying or working in/with at least one language and were selected from cities in different provinces of China with the majority in Anhui province (1,530/91.67%) and other 17 provinces (Fujian, Guangdong, Guangxi, Hainan, Hebei, Henan, Hubei, Hunan, Jiangsu, Jiangxi, Jilin, Shandong, Shanxi, Sichuan, Xinjiang, Yunnan, Zhejiang), three municipalities (Beijing, Shanghai, Chongqing) and one autonomous region (Ningxia) (139/8.33%). Informed consent and willingness to participate in this study were obtained from all 1,669 participants before they participated voluntarily via WeChat utilizing convenience sampling.

### Instruments

#### Five Facets of Mindfulness Questionnaire

The FFMQ by Baer et al. (2006) was utilized to estimate learners' mindfulness, operationalized by five aspects: monitoring, explaining, behaving with recognition, and being unbiased regarding internal encounters, and not responding to internal encounters. This survey involved 39 declarations meant to signify these various dimensions. The subjects were asked to allocate a number that resembled the truth of each sentence that applied to them. It should be noted that its reliability was calculated by Cronbach's Alpha and it was 0.92.

#### Resilience Scale

Built by Lereya et al. (2016), the resilience scale is a 40-item standard incorporating 12 subscales evaluating students' insights of their specific attributes as well as protecting dimensions fixed in the context. The rate of each item was measured on a 5-point scale. It should be noted that the consistency of the questionnaire was calculated by Cronbach's Alpha and it was 0.97.

#### Student Engagement Instrument

A 35-item self-document standard of learner engagement, the SEI was built from a large pool of items that were separated into 35 through exploratory and CFAs (Appleton et al., 2006). The SEI questionnaire item answer options were contingent on a 4-point Likert-similar scale (from strongly disagree to strongly agree). The SEI is believed to estimate three sub-kinds of affective engagement (i.e., educator-learner connections, colleague help for studying, guardian help studying) and three sub-kinds of intellectual engagement (regulation and significance of school tasks, future objectives and dreams, and innate inspiration). It is worth noting that its reliability was calculated by Cronbach's Alpha and it was 0.97.

### Data Collection Procedures

To meet the objectives of the study, by distributing questionnaires online, data were collected at the end of January *via* the WeChat phone app through Wenjuanxing (an online questionnaire program to collect data). To enhance the generalizability of the results of this study, data were gleaned from various provinces, municipalities, and autonomous regions in China. They were notified that they would put themselves into “normal times” when answering the statements in the questionnaire rather than the COVID-19 pandemic. In order to increase the validity and reliability of the sample, all participants were informed of how to fill in the questionnaire and assured that their responses and personal information would only be used for research purposes and would remain confidential. Then, the collected data were sent to SPSS software for further analysis. In the final step, the exploration of the research questions was conducted based on the processed data.

### Data Analysis

To study the plausible correlation among the key terms of the study, whether there is a relationship between Chinese EFL students' mindfulness, resilience, and engagement, the Spearman Rho test was used. In addition, for the second research question which intends to examine the predictor role of mindfulness and resilience in learners' engagement, a linear multiple regression analysis was run.

## Results

The purpose of the study is to scrutinize the role of language learners' academic resilience and mindfulness on their engagement. To make sure of the reliability of the instruments used for data collection, the researcher ran a Cronbach Alpha test for each questionnaire.

Table 1 shows that all three questionnaires including students' mindfulness, resilience and engagement had satisfactory Cronbach Alpha indices (0.92, 0.97, and 0.97, respectively).

**Table 1:** Reliability of the questionnaires.

**Questionnaires**	**Cronbach's alpha**	***N* of items**
Mindfulness	0.92	24
Resilience	0.97	40
Engagement	0.97	35

After making sure of the reliability of the questionnaires, the researcher ran a normality test to decide whether the data should be analyzed parametrically or not. Table 2 depicts that the collected data were not normal for all of the variables, since *P* value for them are (students' mindfulness, resilience, and engagement) 0.000. Thus, it violated the assumption of normality and the data had to be analyzed non-parametrically using a Spearman Rho correlation index.

**Table 2:** Test of normality.

	**Kolmogorov-Smirnov** ^a^			**Shapiro-Wilk**		
	**Statistic**	**df**	**Sig**.	**Statistic**	**df**	**Sig**.
Mindfulness	0.111	1,669	0.000	0.945	1,669	0.000
Resilience	0.087	1,669	0.000	0.967	1,669	0.000
Engagement	0.203	1,669	0.000	0.877	1,669	0.000

a *Lilliefors significance correction*.

### The First Research Question

The first research question concerns the existence of any relationship between Chinese EFL students' mindfulness, resilience, and engagement. To this end, after making sure that the data are not normal, a Spearman Rho test was run.

The first research question concerns the existence of any relationship between Iranian EFL students' mindfulness, resilience and engagement. Since it was proven that the data are not normal in the previous section, a Spearman Rho test was run.

Spearman Rho index shows the amount and the direction of the relationship among the variables. Table 3 demonstrates that the relationship between students' engagement and the other variables (mindfulness and resilience) are direct (0.31, 0.59), which means that the higher index of students' engagement, the higher indices of the other variables. Furthermore, the significance level for all of these relationships are 0.000, which means that there is a direct and significant relationship among the variables of the study.

**Table 3:** Correlations among students' mindfulness, resilience and engagement.

		**Mindfulness**	**Resilience**
Engagement	Correlation coefficient	0.31	0.59
	Sig. (2-tailed)	0.000	0.000
	*N*	1,669	1,669

### The Second Research Question

The second research question deals with the extent to which Chinese EFL students' mindfulness and resilience can predict their engagement. To measure this prediction, a linear multiple regression analysis was performed.

Table 4, the model summary, shows that much of the variance in the dependent variable (scores obtained from engagement) can be explained by the model (which included the variables of students' mindfulness and resilience). In this case, the value for *R*^2^ equals 0.507). Expressed as a percentage, it implies that the model (which included scores on students' mindfulness and resilience) explained 50 percent of the variance in scores from students' engagement.

**Table 4:** Model summary for students' mindfulness, resilience and engagement.

**Model**	***R***	***R* square**	**Adjusted**	**Std. error of**
			***R* square**	**the estimate**
1	0.712	0.507	0.506	11.424

To evaluate the numerical significance of the findings, it was essential to look at Table 5 named ANOVA. This verified the premise that multiple R in the sample equals zero (0). The model extended statistical meaning [*F* = (2, 1,666) = 855.18, Sig = 0.000, this really means *p* < 0.05].

**Table 5:** ANOVA for students' mindfulness, resilience and engagement.

**Model**		**Sum of**	**df**	**Mean**	***F***	**Sig**.
		**squares**		**square**		
1	Regression	223236.38	2	111618.19	855.18	0.000
	Residual	217446.54	1,666	130.52		
	Total	440682.93	1,668			

Although the data were not normal, performing a linear multiple regression analysis seems to be an efficient way to measure the predictability power for large sample size. To identify which of the variables involved in the model contributed more to the prediction of the dependent variable; the researcher checked the column labeled “Beta” in Table 6. To associate the various variables, it was felt necessary to look at the *standardized* coefficients, not the *unstandardized* ones. “Standardized” means that these values for each of the different variables have been converted to the same scale so that one can compare them.

**Table 6:** Coefficients for students' mindfulness, resilience and engagement.

**Model**		**Unstandardized**		**Standardized**	***t***	**Sig**.
		**coefficients**		**coefficients**		
		***B***	**Std. error**	**Beta**		
1	(Constant)	50.89	1.55		32.76	0.000
	Mindfulness	0.011	0.020	0.215	0.55	0.011
	Resilience	0.405	0.011	0.716	37.51	0.000

In this study, the researcher was interested in *comparing* the contribution of each independent variable; therefore, the beta values are used. Looking down the Beta column, she found that the largest beta coefficient was 0.71, which was for students' resilience. This indicates that this variable made the robust distinctive support to clarifying the dependent variable when the variance clarified by all other variables in the model was controlled. The Beta value for learners' mindfulness (0.21) was also significant since the Sig value was 0.011, which was <0.05.

## Discussion

The purpose of the current research was to promote one's knowledge of the association among resilience, mindfulness, and engagement. The outcomes proved the strong relations among all three variables with mindfulness and resilience meaningfully predicting learners' engagement. The present study specified two essential predictors of engagement in EFL students' English learning and increased the empirical proof of the constrained mindfulness and resilience literature in educational environments. These results support previous research, which found that mindfulness upsurges learners' engagement (Meiklejohn et al., 2012) and resilience predicts engagement (Ahmed et al., 2018). The results of the present study support those of Palmer and Rodger (2009) who anticipated that mindfulness may upsurge one's capability to tackle tension which means those with a high degree of mindfulness scored meaningfully lower on the tension and stress. It can be stated that by practicing mindfulness, learners have the prospective to improve reflexivity along with abilities to help them regulate their stress and consequently maintain their engagement. The results are in line with Davidson and Begley (2012) who have specified in their research that mindfulness can enhance the recognition of one's beliefs, emotions, and actions, and enhance focus, intellectual regulation, and emotive control. As it was shown that the greatest predictor of English learning engagement is mindful learning, the results were consistent with the preceding studies indicating the relationship among mindful learning, learning function, and grades (Bakosh et al., 2016). Mindfulness can also release tension, decrease apprehension and hopelessness, develop a constructive mood, lessen destructive attitudes, and boost societal, emotive, behavioral, and physical health consequences. A person who is mindful of education concentrates on the current moment, is interested in various methods of investigating knowledge, attaining information, and therefore, dynamically engaging in the educational cycle (Langer, 2016). Moreover, mindfulness aims at assisting the person to understand facts in a better and more obvious manner that also assists learners to recognize themselves and improve their educational degree. Low level of mindfulness allows the brain to search and concentrate on the stressing factors, resulting in the intensification of the negative affective response and therefore impeding resilience. The findings of the study are congruent with the one carried out by Artika et al. (2021) who demonstrated mindfulness predicts learners' engagement and mindfulness is highly critical to contribute to renewing learners' cognition and affection and reinforce their engagement while learning. It is represented in tasks involving fun learning which possibly allows learners have a higher sense of comfort, lower fear, and pleasance during studying. That is, students with mindfulness will have greater fun with the process of learning, which then makes students have engagement and higher function in learning.

Since there is a relationship between mindfulness and resilience in learners, then increasing interferences and treatments that target developing learners' mindfulness may be advantageous in the reinforcement of resilience in the language process. The resilience-student engagement relationship is mentioned in several investigations because learners with lower levels of resilience had lower engagement levels as well (Pidgeon and Keye, 2014). Based on the results, mindfulness is a positive and significant predictor of resilience and is consistent with the research conducted by Meiklejohn et al. (2012), stating that educating mindfulness significantly increases the resilience level. It means that mindfulness requires being resilient, and once people are mindful, they easily accept changes, can adapt to new situations, and have perseverance in following activities. The manner in which learners encounter issues in the class must be explored since it is a component of learners' educational resilience. Certainly, educational resilience in learners must be contemplated since low educational resilience can result in unease and a hard time dealing with issues, which emerge from school issues. This hardship can result in learners participating less in classes and it describes how educational resilience can affect learners' participation in the setting of language education. In addition, Keye and Pidgeon (2013) also designated that mindfulness can improve learners' resilience. Through mindful learning, psychological assets are linked to the achievement of dynamic engagement in language learning, and the impact of mindful learning is significant in the alteration from cognitive attentiveness to the developmental actualization of language learning commitment. That is, a person who is effective, expectant, positive, and resilient is more prone to take pleasure in attaining novel information and to dynamically engage in the educational cycle when he or she also concentrates on and recognizes the educational cycle.

## Conclusion and Implications

The upshots of this study have implications for educators. To develop learners' educational presentation, they should not only emphasize the learning but also deliberately develop and increase learners' inner constructive abilities such as resilience, and engagement. They should develop their learners' psychological resilience and learning engagement that aids them to be able to face the difficulties in education and lifespan, and sequentially expand their academic success. Moreover, these days, it is mentioned that mindful attention abilities should be learned in school as a global program targeted toward prohibition, because they have the advantage for all learners through reducing tension, enhancing social competency, and are related to enhancing educational function, that may enhance self-efficacy, construct resilience, and improve learner engagement within the classroom (Schonert-Reichl et al., 2015).

Educators and learners can also enjoy the inclusion of mindfulness within the academic environment, because mindfulness training can assist educators to deal with their tension and, consequently, can have an impact on their interference with students (Gold et al., 2010). Mindful students who display lively participation and excessive inquisitiveness, in addition to a recognition of various opportunities and views for carrying out assignments and an attendance to what is occurring in the current state, incline to be more involved and participate more in educational exercises (Schreiner and Louis, 2006). Additionally, proof from past studies has demonstrated that mental or psychological capital (PsyCap) and mindful education are successful in enabling and promoting educational results (Bakosh et al., 2016). With the help of mindful education, a person focuses on the educational cycle, realizes his or her educational habits and manners, and looks for substitute solutions when possible (Langer, 2016). Mindful education, which enables people to concentrate more on what is being learned, is related to constructive educational encounters and ends in better educational results. Based on the findings of the present research, there is support for growing plans for language learners that aims to foster resilience and mindfulness that lead to the enhancement of their capability to efficiently control the difficulties that assist their engagement. The findings can be very useful to EFL students, as being mindful and resilient, encourages and permits them to be more objective-directed and effective in studying a foreign language.

Since English in China is studied as a foreign language and not in a normal English-speaking setting, it is harder for students to attain the language normally and wholly. Thus, English language education necessitates students to try more, sensibly focus more on the conventional attributes of the language, and dynamically take part in effectively attaining English language abilities. Mindful education assists students with recognizing the educational environment more and being more engaged in education. As the significant help of constructive effect of mindfulness, it can be concluded that it can enhance engagement and well-being and consequently grow students with high levels of engagement. English language teachers can execute the outcomes of mindfulness-relevant research in their classes and can train mindfulness which results in more engagement. Materials developers can plan reading materials and assignments contingent on mindfulness-related guidance to improve learners' engagement and assist them in learning in a suitable class setting. This is because students can exercise mindfulness training with sufficient training.

The current paper only involved English language, college learners; nonetheless, the paucity of research in other realms and future studies in education is necessary to scrutinize the association among several aspects beneficial to learning at various steps of language learning and private language schools and also among English educators. Moreover, the scholars in this research examined the relationships among the factors in a quantitative manner. Both quantitative and qualitative approaches can be implemented to carry out more meticulous studies. In regard to the result that mindfulness is significantly related to resilience, it is suggested that future research employ experimental approaches to repeat those outcomes to get a more complete comprehension of those links and their implications for the constructive prospect of psychological wellbeing. In addition, like other research, the present study was done with specific limitations and constraints. The researcher had no access to an identical number of male and female subjects; therefore, gender can be an interfering element in the present research. Consequently, it is recommended to conduct a research where gender could be under control. In regard to the whole evidence supporting the mindfulness advantages and the education capability to increase the mindfulness quality, the present study determined that educating mindfulness can be employed as an alternative interference that can assist learners to enhance their involvement and enhance their capability to succeed in learning English.

## Data Availability Statement

The original contributions presented in the study are included in the article/supplementary material, further inquiries can be directed to the corresponding author/s.

## Ethics Statement

The studies involving human participants were reviewed and approved by China University of Geosciences Academic Ethics Committee. The patients/participants provided their written informed consent to participate in this study.

## Author Contributions

All authors have made a direct and intellectual contribution to the work and approved it for publication.

## Conflict of Interest

The authors declare that the research was conducted in the absence of any commercial or financial relationships that could be construed as a potential conflict of interest.

## Publisher's Note

All claims expressed in this article are solely those of the authors and do not necessarily represent those of their affiliated organizations, or those of the publisher, the editors and the reviewers. Any product that may be evaluated in this article, or claim that may be made by its manufacturer, is not guaranteed or endorsed by the publisher.
